# The Best in CytoJournal: 2006

**DOI:** 10.1186/1742-6413-4-12

**Published:** 2007-06-11

**Authors:** Michael B Cohen

**Affiliations:** 1 Department of Pathology, 200 Hawkins Drive, C670 GH, The University of Iowa, Iowa City, IA, USA

## Abstract

This year CytoJournal celebrates the second full year of publication with its Best of CytoJournal: 2006. Like last year, it recognizes the work of the authors in thyroid FNAB. And, importantly, this years award, which was handed out at USCAP, was endowed by a gift from the Pathikonda family.

## Background

In 2005 CytoJournal established a "Best of..." award and we were pleased to recognize the work of Nguyen, et al. [1]. For 2006 we are again most pleased to recognize another set of authors, led by Dr. Deveci [2] after evaluating all CytoJournal articles published in 2006 [5-28]. In addition, this year's award is a named award, the Pathikonda Award, and is supported by a generous gift from Pathikonda family. Finally, I would like to thank the reviewers who kindly contributed their time to help select this year's winner.

Interestingly, both articles are about thyroid fine-needle aspiration biopsy (FNAB). And, like last year, the article by Deveci et al. is, notably, one of the 'highly accessed' articles during the past year [3]. As I stated last year, thyroid FNAB is a very useful way of evaluating and managing such nodules. In fact, it is often the initial triage point [4]. The review by Drs. Nguyen, et al. provided a succinct discussion of the cytologic features that can be encountered by cytologists in evaluating thyroid FNABs, including the utility of ancillary studies [1]. The paper by Deveci et al. complements that review with an examination of an institution's experience with follicular lesions of the thyroid [2].

The award presentation was made by Dr. Vinod Shidam, the journal's Executive editor and Co-Editor in Chief, at the just past USCAP meeting in San Diego, and can be viewed at: http://www.cytopathology-foundation.org/page12.html.

In closing, we congratulate the authors on their article and hope that at the same time this will interest other authors in submitting their articles to CytoJournal.
